# Changes in Key Biodiversity Area networks following national comprehensive assessments

**DOI:** 10.1111/cobi.70151

**Published:** 2025-09-30

**Authors:** Andrew J. Plumptre, Zoltan Waliczky, Daniele Baisero, Olivia Crowe, Jeannot Kivono, Cecilia Tobar, Maria Gabriela Toscano, Natalia Boulad, Hugo Costa, Camila Davila, Sophie Dirou, Eleuterio Duarte, Karolina Fierro, Carolina Castellanos‐Castro, Hanna Haddad, Stephen Holness, Fiona Maisels, Daniel Marnewick, Menard Mbende, Maitha Abdulla Al Mheiri, Dissondet Moundzoho, Simon Nampindo, Grace Nangendo, Steeve Ngama, Catherine Numa, Diego Peñaranda, Samridhi Rijal, Manuel Sánchez‐Nivicela, Andrew Skowno, Thomas Starnes, Nicolas Texier, Lize von Staden, Anne Bowser, Thomas M. Brooks, Gill Bunting, Stuart H. M. Butchart, Neil Cox, Wendy Elliot, Jo Gilbert, Penny Langhammer, Olivier Langrand, Rachel Neugarten, Madhu Rao, Jon Paul Rodriguez, Gina della Togna, Amy Upgren, Stephen Woodley

**Affiliations:** ^1^ KBA Secretariat, c/o BirdLife International Cambridge UK; ^2^ Conservation Science Group, Department of Zoology Cambridge University Cambridge UK; ^3^ BirdLife International Cambridge UK; ^4^ WWF, Kinshasa, Democratic Republic of the Congo Goma Democratic Republic of the Congo; ^5^ IUCN Regional Office for West Asia Amman Jordan; ^6^ Wildlife Conservation Society Bronx New York USA; ^7^ ECOAN Cusco Peru; ^8^ Instituto de Investigación de Recursos Biológicos Alexander von Humboldt Bogotá Colombia; ^9^ Centre for African Conservation Ecology & Institute for Coastal and Marine Research Nelson Mandela University Gqeberha South Africa; ^10^ Faculty of Natural Sciences University of Stirling Stirling UK; ^11^ IUCN Eastern and Southern Africa Regional Office Johannesburg South Africa; ^12^ BirdLife South Africa Johannesburg South Africa; ^13^ Ministry of Climate Change and Environment Dubai United Arab Emirates; ^14^ Missouri Botanical Garden St. Louis Missouri USA; ^15^ Gembloux ABT University of Liège Liège Belgium; ^16^ PFS‐DD, IRAF Centre National de la Recherche Scientifique et Technologique Libreville Gabon; ^17^ IUCN Centre for Mediterranean Cooperation Campanillas Spain; ^18^ Asociación Armonía Santa Cruz de la Sierra Bolivia; ^19^ Fundación de Conservación Jocotoco Quito Ecuador; ^20^ Instituto Nacional de la Biodiversidad (INABIO) Quito Ecuador; ^21^ Laboratorio de Biología Evolutiva ‐ USFQ Quito Ecuador; ^22^ South African National Biodiversity Institute Cape Town South Africa; ^23^ Biological Sciences University of Cape Town Cape Town South Africa; ^24^ IUCN Cambridge UK; ^25^ Faculty of Sciences, Evolutionary Biology and Ecology Université Libre de Bruxelles Brussels Belgium; ^26^ NatureServe Arlington Virginia USA; ^27^ IUCN Gland Switzerland; ^28^ World Agroforestry Center (ICRAF) Nairobi Kenya; ^29^ ICRAF University of The Philippines Los Baños Laguna Philippines; ^30^ Institute for Marine & Antarctic Studies University of Tasmania Hobart Tasmania Australia; ^31^ Department of Zoology University of Cambridge Cambridge UK; ^32^ Conservation International Washington, DC USA; ^33^ WWF International Nairobi Kenya; ^34^ Royal Society for the Protection of Birds Sandy UK; ^35^ Re:Wild Austin Texas USA; ^36^ Critical Ecosystem, Partnership Fund Washington, DC USA; ^37^ World Commission on Protected Areas, IUCN Gland Switzerland; ^38^ IUCN Species Survival Commission Gland Switzerland; ^39^ Amphibian Survival Alliance Austin Texas USA; ^40^ American Bird Conservancy Washington, DC USA

**Keywords:** biodiversity assessment, conservation planning, Convention on Biological Diversity, Key Biodiversity Area, Kunming–Montreal Global Biodiversity Framework, Área Clave para la Biodiversidad, Convenio sobre la Diversidad Biológica, evaluación de la biodiversidad, Marco Mundial de Biodiversidad Kunming‐Montreal, planeación de la conservación, 关键生物多样性区域(KBA), 生物多样性评估, 《生物多样性公约》, 《昆明-蒙特利尔全球生物多样性框架》, 保护规划

## Abstract

Key Biodiversity Areas (KBAs) are sites of significance for the global persistence of biodiversity. Based on the Global Standard for the Identification of Key Biodiversity Areas (KBA Standard), published in 2016, sites are currently being assessed for KBA designation in a growing number of countries across the world. For these assessments, the KBA criteria are applied to all species and ecosystems with available data. We reviewed the first comprehensive assessments of 11 countries and compared the KBA network before and after assessments. The mean (SD) number of KBAs per country increased by 69.6% (102.1), and the mean total extent of KBAs per country increased by 164.2% (150.7). More than half of the KBAs in 2024 had >50% of their area outside the 2019 KBAs, indicating a substantial increase in KBA extent (54.0% [18.8] of KBAs). The mean proportion of each KBA covered by protected or conserved areas decreased from 56.2% (20.2) to 44.5% (15.5), owing to the incorporation of unprotected sites in the KBA network. On average, 41.1% (14.0) of sites in each country (mean 44.5 [46.4] sites per country) and 47.2% (20.5) of new KBA area after the assessment were completely unprotected, indicating that many of the new sites were not recognized in national protected area networks as significant for biodiversity before the assessment. Making a comprehensive assessment of KBAs increased the combined coverage of protected and conserved area networks from 25.4% (10.6) to 32.0% (13.1) in each country and thus contributed to reducing biodiversity loss. Therefore, comprehensive assessments of KBAs led to a substantially increased number and extent of recognized sites of importance for biodiversity published in the World Database of KBAs. Where such assessments have not been made, many important areas for biodiversity may be overlooked. We therefore encourage other nations to update their KBA networks to inform efforts to meet the goals and targets of the Kunming–Montreal Global Biodiversity Framework.

## Graphical abstract

Making comprehensive assessments of Key Biodiversity Area (KBA) networks in a country greatly increases their number and area. Countries that have not yet done so will be missing about half of these globally significant sites and missing the opportunity to get 30 × 30 conservation in the right places.

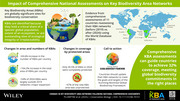

## Abstract (es)

Cambios en las redes de Áreas Clave para la Biodiversidad después de una evaluación nacional completa

**Resumen:**

Las áreas clave para la biodiversidad (ACB) son sitios de importancia para la persistencia global de la biodiversidad. Con base en la Norma Global para la Identificación de Áreas Clave para la Biodiversidad (Norma ACB), publicada en 2016, hoy en día se evalúan sitios para su designación como ACB en un número cada vez mayor de países de todo el mundo. Para estas evaluaciones, los criterios ACB se aplican a todas las especies y ecosistemas con datos disponibles. Revisamos las primeras evaluaciones exhaustivas de 11 países y comparamos la red de ACB antes y después de las evaluaciones. La media del número de ACB por país aumentó un 69.6% (102.1) y la extensión total media de las ACB por país aumentó un 164.2% (150.7). Más de la mitad de las ACB en 2024 tenían más del 50% de su superficie fuera de las ACB de 2019, lo que indica un aumento sustancial de la extensión de las ACB (54.0% [18.8] de las ACB). La proporción media de cada ACB cubierta por áreas protegidas o conservadas disminuyó del 56.2% (20.2) al 44.5% (15.5), debido a la incorporación de sitios no protegidos en la red de ACB. En promedio, el 41.1% (14.0) de los sitios de cada país (media de 44.5 [46.4] sitios por país) y el 47.2% (20.5) de la nueva superficie de ACB tras la evaluación carecían por completo de protección, lo que indica que muchos de los nuevos sitios no habían sido reconocidos en las redes nacionales de áreas protegidas como significativos para la biodiversidad antes de la evaluación. La realización de una evaluación exhaustiva de las ACB aumentó la cobertura combinada de las redes de áreas protegidas y conservadas del 25.4% (10.6) al 32.0% (13.1) en cada país, lo que contribuyó a reducir la pérdida de biodiversidad. Por lo tanto, las evaluaciones exhaustivas de las ACB dieron lugar a un aumento sustancial del número y la extensión de los sitios reconocidos como importantes para la biodiversidad publicados en la Base de Datos Mundial de ACB. Cuando no se han realizado tales evaluaciones, es posible que se ignoren muchas áreas importantes para la biodiversidad. Por lo tanto, alentamos a otras naciones a que actualicen sus redes de ACB para informar los esfuerzos destinados a cumplir los objetivos y metas del Marco Mundial de Biodiversidad de Kunming‐Montreal.

## 国家综合评估后关键生物多样性区域网络的变化

**【摘要】**关键生物多样性区域(Key Biodiversity Area, KBA)是对全球生物多样性续存具有重要意义的位点。目前世界上越来越多的国家正在基于2016年发布的《全球关键生物多样性区域识别标准》(KBA标准), 对被认定为KBA的位点进行评估。这些评估在所有具有可用数据的物种和生态系统中应用了KBA标准。本研究回顾了11个国家的首次全面评估, 并对评估前后的KBA网络进行了比较。我们发现, 每个国家的KBA平均数量增加了69.6%(标准差为102.1), KBA总面积平均增加了164.2%(标准差为150.7)。在2024年的KBA中, 超过一半有>50%的区域在2019年的KBA之外, 表明KBA的范围大幅扩张(54.0%[18.8]的KBA)。由于有未受保护的位点被纳入KBA网络, 因此每个KBA被自然保护地覆盖的平均比例从56.2%(20.2)降至44.5%(15.5)。平均而言, 每个国家有41.1%(14.0)的位点(平均44.5[46.4]个)和评估后47.2%(20.5)的新KBA完全未得到保护, 这表明许多新位点在评估前未被国家自然保护地网络认定为对生物多样性具有重要意义。对KBA进行全面评估后, 各国自然保护地网络的总覆盖率从25.4%(10.6)增加到了32.0%(13.1), 这有助于减少生物多样性丧失。因此, 对KBA的全面评估使世界KBA数据库中公布的被认为对生物多样性具有重要意义的位点的数量和面积都大幅增加。如果没有进行此类评估, 许多对生物多样性具有重要意义的地区可能会被忽视。因此, 我们鼓励其他国家更新其KBA网络, 从而为实现《昆明‐蒙特利尔全球生物多样性框架》的目标和指标提供信息。**【翻译:胡怡思;审校:聂永刚】**

## 1 INTRODUCTION

In December 2022, the world's governments came together to adopt a new plan to reverse biodiversity loss: the Kunming–Montreal Global Biodiversity Framework (KMGBF). Participating governments were required to revise and update their national biodiversity strategies and action plans (NBSAPs) to reflect the 4 goals and 23 targets of the framework. The KMGBF aims to halt human‐induced extinction of species and reverse biodiversity loss to put nature on a road to recovery by 2030. Target 21 in the framework aims to “ensure that knowledge is available, accessible and used to guide biodiversity action” (CBD, 2022), and most of the other targets rely on data to guide actions needed to meet the targets. Targets 1 and 3 promote national spatial planning for biodiversity and expansion of protected and conserved areas (other effective area‐based conservation measures [OECMs]), respectively. Target 3 includes a commitment to cover 30% of land and seas with protected or conserved areas by 2030 (i.e., 30×30 target). Notably, Targets 1 and 3 identify the need to focus on “areas of particular importance for biodiversity.” Biodiversity data are essential to identify and delineate these areas (Plumptre, Baisero, et al., 2024), and the process of compiling biodiversity information in the identification of such areas contributes to achieving Target 21 and Targets 1 and 3. A number of approaches have been used to identify sites of biodiversity importance, including Important Bird and Biodiversity Areas (IBAs) (Donald et al., 2019), Important Plant Areas (Darbyshire et al., 2017), Important Marine Mammal Areas (Tetley et al., 2022), Important Shark and Ray Areas (Hyde et al., 2022), and Alliance for Zero Extinction sites (AZEs) (Ricketts et al., 2005). Most of these approaches focus on particular subsets of species, so the Key Biodiversity Area (KBA) concept and criteria (defined in a KBA Standard) (IUCN, 2016) were developed to unify these approaches under a single system and enable “sites of significance for the global persistence of biodiversity” to be identified for any macroscopic species, ecosystem, or location of high ecological integrity or irreplaceability (IUCN, 2016).

The KBA criteria include quantitative thresholds to allow consistent assessment of whether a site holds a globally significant population of a species, significant extent of an ecosystem, or outstanding ecological integrity or irreplaceability at a global level (Appendix S1). They identify globally significant sites because the criteria contain thresholds for the proportion of the global population size of a species or the global extent of an ecosystem (Appendix S1). The identification of a site as a KBA implies that the site should be managed in ways that ensure the persistence of the biodiversity elements for which it is important (IUCN, 2016) but is not prescriptive about the means of such management, which depends on the specific situation at each site (Smith et al., 2019). Many sites are best conserved through formal protected areas, whereas others may be best managed through OECMs (conserved areas) or other approaches.

Because the application of quantitative criteria means that sites are comparable (e.g., between countries) (Plumptre, Baisero, et al., 2024), KBAs can form the basis of indicators for use in multilateral environmental agreements. Trends in the coverage of KBAs by protected and conserved areas are used by the United Nations to track progress in sustainable development goals 14 and 15 (specifically, indicators 14.5.1 ‐Marine; 15.1.2 ‐ terrestrial and freshwater; 15.4.1 ‐ mountain KBAs, respectively) (United Nations, 2025) and by the Convention on Biological Diversity as an indicator for Target 3 of the KMGBF (specifically, as a recommended disaggregation of headline indicator 3.1 on coverage of protected and conserved areas) (CBD, 2024). In various countries, KBAs are also being used to guide conservation planning and support land‐ and sea‐use planning. They are increasingly informing decisions made by private sector organizations to minimize and disclose their biodiversity risks and are also used by a growing number of donors to guide their investments in conservation.

There are over 16,500 KBAs confirmed in the World Database of KBAs (BirdLife International, 2025). Many of these sites were originally identified as IBAs (Donald et al., 2019) or AZEs (Ricketts et al., 2005) or were KBAs identified through ecosystem profiles for biodiversity hotspots commissioned by the Critical Ecosystem Partnership Fund (CEPF) (CEPF, 2023) based on prototype criteria (Langhammer et al., 2007). Prior to the first comprehensive KBA assessments following the publication of the KBA Standard, IBAs comprised almost 85% of KBAs (BirdLife International, 2019), and CEPF KBAs comprised 37% and AZEs 5% (there is an overlap across these groups). The KBA criteria have since been consolidated and expanded into a formal KBA Standard; hence, these KBAs now need reassessing against the new KBA Standard (IUCN, 2016) to identify which qualify as global KBAs (meeting the global KBA criteria) and which qualify as regional KBAs (meeting previously established criteria and thresholds), as described in the KBA Standard (IUCN, 2016). At present 35.8% of the sites in the World Database of KBAs have been demonstrated to meet global criteria (BirdLife International, 2025). Following the adoption of the KBA Standard in 2016, biodiversity experts and institutions across the world have begun to apply these criteria to existing KBAs and use them to identify additional KBAs. To date, 41 countries have established KBA national coordination groups to bring together stakeholders across government, civil society, academia, Indigenous Peoples’ groups, and other sectors to undertake national assessments of KBAs across multiple taxonomic groups of species, ecosystems, and sites of ecological integrity or irreplaceability (Plumptre, Baisero, et al., 2024).

Identifying a site as a KBA can bring it local, national, regional, and global attention but does not guarantee its conservation. Although there has not yet been an assessment of the impact of identification of sites as KBAs on their conservation and biodiversity outcomes, some studies have focused on subsets of KBAs. Species occurring in IBAs or AZEs with greater coverage by protected areas experienced smaller increases in extinction risk (shown by the International Union for Conservation of Nature Red List Index) over recent decades. The increase was half as large for bird species; >50% of the IBAs at which they occur are completely covered by protected areas. The increase was a third lower for birds, mammals, and amphibians restricted to protected AZEs (compared with unprotected or partially protected sites) (Butchart et al., 2012). Trends in wintering waterbird abundance across Europe and North Africa during 1990–2015 were consistently more positive inside IBAs than other wetlands (Pavón‐Jordán et al., 2020). Across Australia, southern Africa, and Europe, IBAs have higher irreplaceability on average than other sites (Di Marco et al., 2016). As a result of these and other studies, we conclude that KBAs are useful in planning protected area expansion because they identify sites with species of conservation concern (Kullberg et al., 2019).

Although virtually all countries have existing KBA inventories (only Monaco, San Marino, and Tuvalu do not [BirdLife International, 2025]), few are comprehensive, drawing on all available biodiversity data to apply as many of the KBA criteria as possible across all taxonomic groups, ecosystems, and sites of ecological integrity. Most existing KBAs have been identified for birds, and 28.4% of KBAs qualify for taxa other than birds, for the ecosystems they support, or for their ecological integrity or irreplaceability (BirdLife International, 2025). However, many sites have not been assessed for their KBA status based on data on taxa other than birds. Prior to 2016, no KBAs were assessed for their significance for ecosystems, ecological integrity, or irreplaceability. The KBA networks are most useful when site identification considered all taxonomic groups, ecosystems, and criteria because then end users can be more certain that the set of sites is comprehensive.

Eleven countries have completed a relatively comprehensive assessment of their KBAs: Bolivia, Colombia, the Democratic Republic of the Congo, Ecuador, Gabon, Mozambique, Peru, the Republic of the Congo, South Africa, Uganda, and the United Arab Emirates (BirdLife International, 2025). Although this is still a relatively small total, it is useful to assess the impacts on national KBA networks of making comprehensive assessments to better guide other countries. We therefore evaluated how the KBA network in these 11 countries changed following a comprehensive assessment based on existing biodiversity data and assessed the implications for countries that have not made comprehensive assessments of their KBAs. We also considered the implications of the results of these assessments for implementation of the KMGBF. Although we expected that the number of sites would increase after comprehensive assessment, the magnitude of this increase and the proportion of the final network the existing sites comprise were unknown. This information is important to know because it gives an indication of the number of KBAs that are missing from the network in countries where comprehensive assessments have not yet been made. We also evaluated the proportion of the KBA network that was covered by protected and conserved areas to evaluate the extent to which newly identified KBAs were recognized as important sites and to what extent assessments identified new areas.

## 2 METHODS

Conducting a comprehensive assessment of KBAs involves reviewing the data available for all biodiversity elements (species, ecosystems, ecological integrity, and irreplaceability) and assessing these against the criteria in the KBA Standard, adding newly qualifying elements to existing sites, removing elements that no longer meet the thresholds, and potentially revising boundaries based on the new knowledge. Some sites that no longer meet KBA criteria are delisted, and new sites may be identified, including for biodiversity elements that had previously not been assessed. KBAs are delineated as sites that have “the possibility of some type of effective management across the site” (IUCN, 2016; KBA Standards & Appeals Committee of IUCN SSC/WCPA, 2022), and they cannot overlap each other. The aim of the KBA program is to identify sites that can be managed for the biodiversity elements that qualify the site as a KBA, and hence, the manageability of the site is an additional key feature. This manageability of a site typically constrains the size of a given KBA. Larger areas are more likely to contain sufficient proportions of a species’ population or an ecosystem's extent to qualify as KBAs but are less likely to be manageable as individual sites. Although IBAs and KBAs identified as part of CEPF ecosystem profiles are also required to be manageable units, some existing KBAs need to be redelineated using up‐to‐date information on local context.

### 2.1 Numbers and area of KBAs

We first estimated the number of sites and total area of sites to assess how the KBA network changed following comprehensive assessments in a country. We extracted data from the World Database of KBAs (BirdLife International, 2025) on the KBA networks in the 11 countries for 2019 and 2024, from before and after comprehensive assessments were undertaken. In some of these countries, a few existing KBAs had not yet been reassessed because the data to do so were not available, and marine KBAs in South Africa have not been assessed. The data we extracted included the species, ecosystems, ecological integrity, and irreplaceability that met the KBA criteria for each KBA in each country and the boundaries for each KBA. We considered the number of sites and the total extent of the network because some KBAs in 2019 were split into 2 or more KBAs in 2024 and other KBAs in 2019 were merged to form a single one in 2024, thereby changing the number of sites but not necessarily the area covered by them. Given that there were some overlapping KBAs prior to reassessment (a consequence of combining different sources [i.e., IBAs, AZEs, and CEPF KBAs] when the KBA program was established), we used ArcGIS Pro 3.1.3 (ESRI, 2024) to first dissolve overlapping KBAs into one layer for each period and then calculated the areas that were exclusive to the inventories in each of 2019 and 2024 and the areas that were common to both. From this geoprocessing step, we calculated the percentage of the 2024 KBAs that overlapped part of the 2019 KBA network and calculated the percentage of sites with <50% overlap.

We calculated the area of KBAs in the 2 periods in each country and the extent and percentage in terrestrial, freshwater, and marine realms separately for each country. For freshwater, we used the method proposed for headline indicator 3.1 in the monitoring framework for the KMGBF (CBD, 2024). This is used to calculate protected area coverage of inland waters (Lehner et al., 2025) by area and that of rivers and streams (Linke et al., 2019) by length. Percent marine area coverage is based on the KBA and protected area boundaries within the Exclusive Economic Zone (EEZ) boundary for the country (Flanders Marine Institute, 2023)

### 2.2 Protected area and OECM coverage of KBAs

Second, we assessed how the extent of protected and conserved area coverage changed following comprehensive assessments in a country. If most new KBAs identified during a comprehensive assessment were recognized as existing protected areas in a country, then there was less urgency to identify them because they were sites that were already recognized as important for conservation. However, if many new KBAs occurred outside the protected area network, then there was a greater urgency for countries to make comprehensive KBA assessments because these sites of biodiversity importance were unrecognized. After addressing overlaps in KBAs in the 2019 dataset by dissolving them into one layer, we used ArcGIS to calculate the total area of KBAs in each country in 2019 and 2024, the area overlapping between 2019 and 2024, the areas covered exclusively in each of the 2 years, and the areas covered by protected areas or OECMs in each of the 2 years based on the World Database of Protected Areas (WDPA) and World Database of OECMs for 2019 and 2024 (https://www.protectedplanet.net/en). For each period, we calculated the total extent and percent cover of KBAs by protected areas and OECMs. We also calculated the number of KBAs with full coverage (>98%) and those that had no coverage (<2%). We considered both protected areas and OECMs because target 3 of the KMGBF refers to both. However, only 3 of the 11 countries (Colombia, Peru, and South Africa) have identified OECMs and submitted data to the World Database of OECMs, and these may not be complete networks yet. Finally, we calculated the percentage of each country's area that was covered by protected areas and OECMs and estimated by how much this would increase if all KBAs were to be designated as protected areas or recognized as OECMs.

### 2.3 Biodiversity elements qualifying sites as KBAs and KBA criteria used

With a focus on increasing the number of taxonomic groups assessed in a comprehensive assessment, we expected an increase in the number of species and taxonomic groups for which KBAs have been identified. Although most existing KBAs have been identified for their significance for birds, it was not clear how many existing sites would qualify as global KBAs by meeting the global criteria in the KBA Standard, how many of these existing sites of importance for birds would prove to be important for other taxonomic groups, and how many additional sites would be identified for nonbird species, ecosystems, or ecological integrity or irreplaceability.

We calculated the number of taxonomic groups as defined by the KBA Secretariat (https://www.keybiodiversityareas.org/working‐with‐kbas/proposing‐updating/criteria‐tools) and the number of species and threatened species that qualified sites as global KBAs for each country. Although the KBA criteria for ecosystems and areas of high ecological integrity are being used increasingly, further to the publication of the Global Ecosystem Typology (Keith et al., 2022) and assessment of risk of ecosystem collapse (Nicholson et al., 2024), their application has not yet been comprehensive enough to undertake a formal comparison between the periods.

Given that many existing KBAs comprise sites originally identified as IBAs, we compared the subset of the original KBA network that comprised IBAs with the updated KBA network following comprehensive assessment. We therefore calculated the number of IBAs that qualified as global KBAs before and after the comprehensive assessment. However, we found that many sites were redelineated or superseded (i.e., replaced by an overlapping KBA with substantially different boundary) during the comprehensive assessment process, which obscured patterns of changes. We therefore also calculated the percentage of the area of the original IBA network in each country that was covered by the final KBA network with a union calculation in ArcGIS 3.1.3.

Tests of the significance of percent changes from 2019 to 2024 were made with Wilcoxon signed rank tests (Zar, 1984).

## 3 RESULTS

### 3.1 Changes in area and numbers of KBAs

**FIGURE 1:**
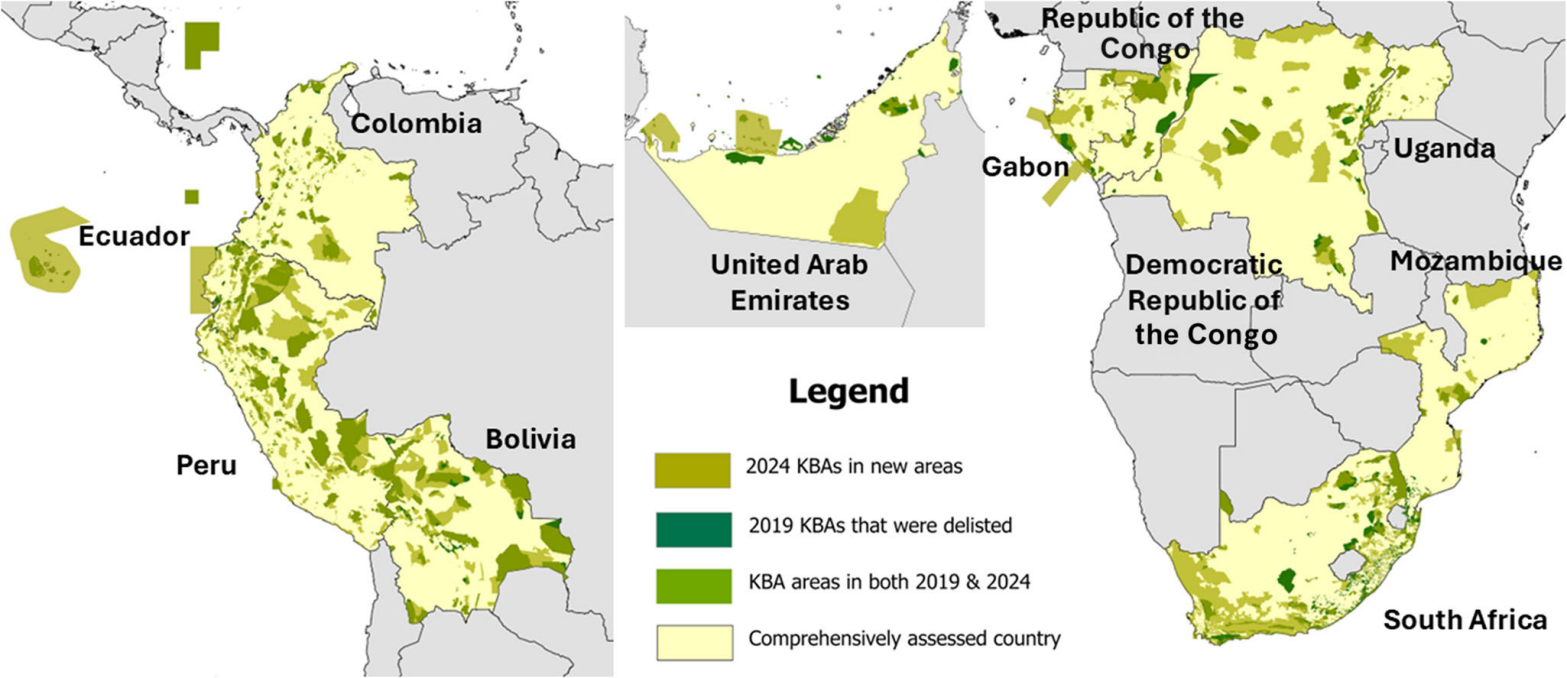
In 11 countries, changes in locations of Key Biodiversity Areas (KBAs) from 2019 to 2024 following comprehensive KBA assessments.

Not surprisingly, following a comprehensive assessment, the number of KBAs increased in most countries (Table 1; Figure 1). There was a decrease in one country, the United Arab Emirates, because some of the existing KBAs did not meet the thresholds established in the KBA Standard (IUCN, 2016) or were merged with other KBAs into larger sites owing to revised judgments about manageability. Other reasons for changes in the number of KBAs in each country were because some sites were split or merged owing to revised information on the distribution of biodiversity or manageability. On average, there was a 69.6% (SD 102.1) increase in the number of KBAs in each country from 2019 to 2024 (Table 1). The mean percentage of KBAs in 2024 that had <50% overlap with the KBA network in 2019 was 54.0% (18.8), indicating that more than half of KBAs in 2024 encompassed substantial new recognized area (Table 1).

**TABLE 1:** Percent increase in the number and total area of Key Biodiversity Areas (KBAs) following comprehensive assessments of KBAs and percentage of 2024 KBAs with <50% overlap with 2019 KBA network.

Country	Change in number of KBAs 2019–2024 (%)	KBAs in 2024 with <50% overlap with 2019 KBAs (%)	Change in KBA area 2019–2024 (%)
Bolivia	54.2	50.5	57.4
Colombia	9.3	36.0	29.8
Republic of the Congo	122.2	50.0	42.7
D. R. Congo^a^	91.7	52.2	128.1
Ecuador	0.8	45.2	299.6
Gabon	337.5	82.9	298.9
Mozambique^a^	66.7	57.1	462.3
Peru	52.3	65.3	100.1
South Africa^b^	57.7	61.9	64.1
Uganda^a^	42.9	15.0	16.6
United Arab Emirates	−70.0	77.8	306.7
Mean (SD)	69.6(102.1)	54.0(18.8)	164.2(150.7)

^a^ Numbers include a few KBAs that have not yet been reassessed.

^b^ South Africa has not yet comprehensively assessed marine KBAs.

A comparison of the total area of KBAs in the 2 periods was a better measure (Figure 1) and showed that the mean extent of KBAs in each country increased by 164.2% (SD 150.7) (Table 1). The mean percentage of the area of each country covered by KBAs increased significantly (Wilcoxon *W* = 0, *p* < 0.005, *n* = 11) from 10.1% (6.4) to 20.1% (8.8) (Table 2). Coverage of land increased from 13.9% (9.9) to 24.6% (13.7) (*W* = 0, *p* < 0.005, *n* = 11), coverage of standing freshwaters from 17.9% (11.1) to 27.3% (17.0) (*W* = 8, *p* < 0.05, *n* = 11), coverage of stream and river length from 13.3% (10.2) to 23.6% (13.8) (*W* = 1, *p* < 0.005, *n* = 11), and coverage of territorial seas (EEZ) from 1.6% (3.4) to 10.3% (9.5) (*W* = 0, *p* < 0.005, *n* = 6) (Appendix S2). Current coverage ranged from 0% of the marine area of the Democratic Republic of the Congo to 55.6% of the terrestrial area of Ecuador (Appendix S2).

**TABLE 2:** Percent coverage of each country (including terrestrial and marine areas in Exclusive Economic Zones) by Key Biodiversity Areas (KBAs) in 2019 and 2024; coverage by protected conserved areas (other effective area‐based conservation measures [OECMs]) in 2024; and total coverage in 2024 by KBAs, protected areas, and OECMs combined.

Country	Coverage by KBAs (%)		Coverage by protected areas and OECMs (%)	Coverage by KBAs, protected areas, and OECMs (%)
	2019	2024	2024	2024
Bolivia	21.2	33.3	27.2	41.5
Colombia	9.0	11.6	31.2	33.4
Republic of the Congo	18.0	25.7	47.2	58.5
D. R. Congo	6.9	15.8	18.7	24.5
Ecuador	7.3	29.2	40.5	50.9
Gabon	6.0	23.9	27.6	31.3
Mozambique	1.9	10.4	19.5	21.3
Peru	10.7	21.3	16.8	28.2
South Africa	17.9^a^	29.4^a^	13.5	22.9
Uganda	8.4	9.7	17.9	19.8
United Arab Emirates	2.6	10.6	19.4	19.6
Mean (SD)	10.1(6.4)	20.1(8.8)	25.4(10.6)	32.0(13.1)

^a^ South Africa has not assessed marine KBAs yet, so only percentage of land is given here.

### 3.2 Changes in coverage by protected areas

**FIGURE 2:**
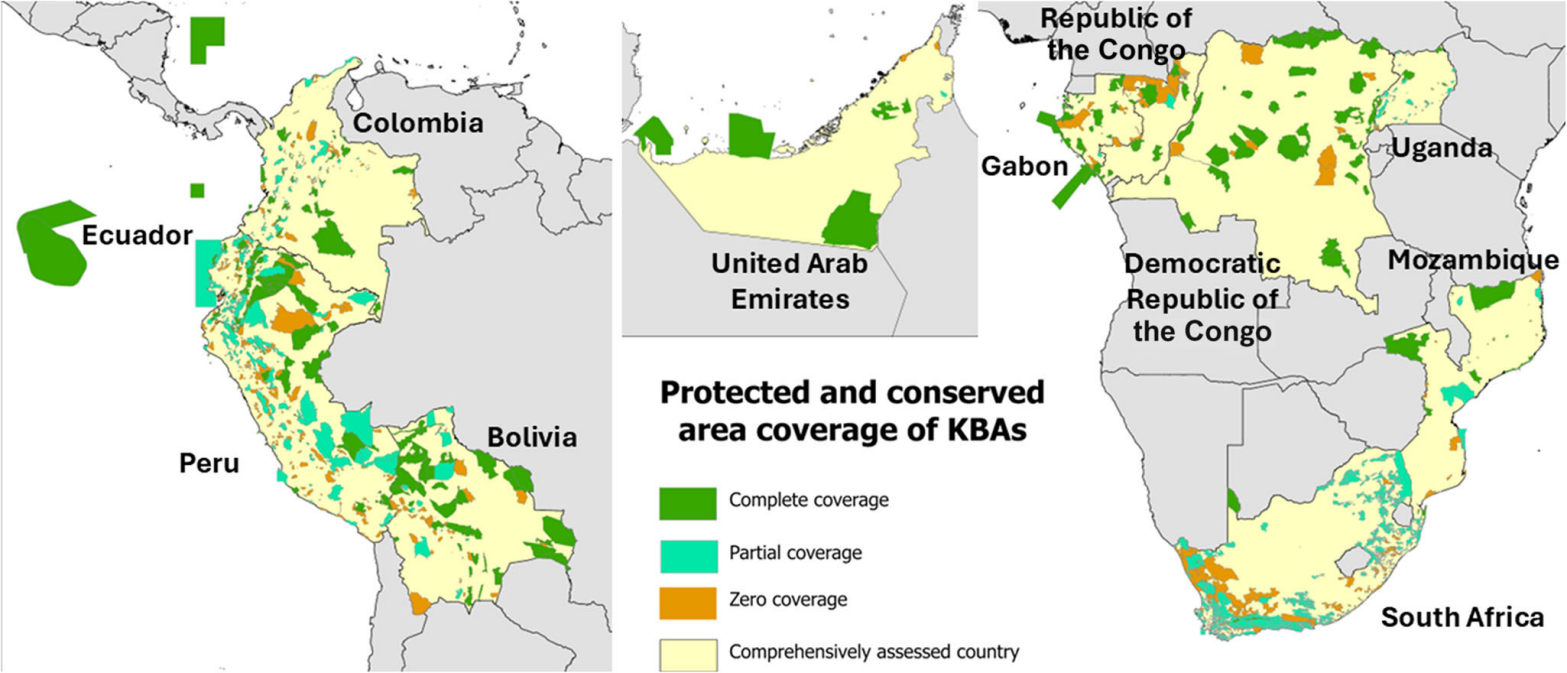
Protected and conserved area coverage of Key Biodiversity Areas (KBAs) in 2024 in the 11 countries where comprehensive KBA assessments were conducted (dark green, KBAs with >98% of their extent covered by protected areas; orange, KBAs with <2% coverage; turquoise, KBAs with 2–98% coverage).

In 2024, the mean proportion of the total extent of KBAs covered by protected and conserved areas showed no change (69.8% [SD 14.4] in 2019 vs. 68.9% [18.0] in 2024) (Figure 2). However, mean coverage of each individual KBA by protected and conserved areas (an official SDG and KMGBF indicator) declined significantly (Wilcoxon *W* = 7, *p* < 0.05, *n* = 11) from 56.2% (20.2) to 45.5% (15.5) in 2024 (Table 3) owing to the addition of unprotected KBAs in 2024. On average, 47.2% (20.5) of the additional area of KBAs identified by 2024 was not covered by protected or conserved areas. Conserved areas contributed a minority of the coverage. In the 3 countries that reported their OECMs, these areas covered a mean of 7.2% (6.0) of each KBA, mirroring the mean coverage of the country by OECMs (7.6% [7.8]). Combined protected and conserved area coverage of the 11 countries averaged 25.4% (10.6) in 2024. If national networks of protected and conserved areas were expanded to cover all KBAs, it would increase the mean coverage of each country to 32.0% (13.1) (Table 2), although complete coverage may not always be desirable or possible.

**TABLE 3:** Coverage of Key Biodiversity Areas (KBAs) by protected and conserved areas before (2019) and after (2024) comprehensive KBA assessments in each country.

Country	KBA extent covered by protected and conserved areas (%)		Mean (%) KBA extent covered by protected and conserved areas		Sites fully covered(%)		Sites with no coverage(%)	
	2019	2024	2019	2024	2019	2024	2019	2024
Bolivia	67.7	57.1	49.5	33.0	20.3	23.1	25.4	48.4
Colombia	79.5	84.9	43.0	66.3	14.0	40.9	30.7	26.2
Republic of the Congo	57.4	55.9	66.3	55.4	30.0	40.0	0.0	30.0
D. R. Congo	74.1	63.0	54.3	53.9	25.0	34.8	29.2	32.6
Ecuador	56.6	64.4	33.2	23.6	12.2	21.0	46.3	66.9
Gabon	90.6	84.6	61.9	56.4	50.0	51.4	12.5	31.4
Mozambique	58.7	82.1	55.5	42.2	19.0	35.1	66.7	51.4
Peru	65.6	47.2	29.2	22.8	16.1	9.7	55.5	62.7
South Africa	46.5	39.6	45.5	31.8	13.6	2.3	36.7	37.0
Uganda	89.2	81.3	84.1	54.6	31.0	21.7	16.7	31.7
United Arab Emirates	81.9	98.1	96.2	60.9	53.3	55.6	40.0	33.3
Mean (SD)	69.8(14.4)	68.9(18.0)	56.2(20.2)	45.5(15.5)	25.9(14.2)	30.5(16.6)	32.7(19.3)	41.1(14.0)

The percentage of KBAs fully covered (>98%) by protected areas or OECMs did not increase significantly over the period (25.9% [SD 14.2] in 2019 vs. 30.5% [16.6] in 2024; Wilcoxon *W* = 19, *p* > 0.05, *n* = 11). Although the percentage of sites that had no coverage (<2% coverage) by protected areas or OECMs increased slightly, this increase was not significant (32.7% [19.3] in 2019 vs. 41.1% [14.0] in 2024; *W* = 14, *p* > 0.05, *n* = 11) (Table 3). Following comprehensive assessment, a mean of 44.5 (46.4) KBAs per country fell outside protected areas and OECMs.

### 3.3 Changes in numbers of species qualifying sites as KBAs

The number of taxonomic groups with at least one species qualifying for KBA criteria (i.e., for which one or more KBAs had been identified) in assessed countries increased significantly (Wilcoxon *W* = 0, *p* < 0.005, *n* = 11) from a mean of 7.9 (SD 9.0) to 25.5 (13.5) after comprehensive KBA assessments (Table 4). The total number of such species increased significantly from a mean of 61.8 (67.0) to 582.5 (1013.0) species (*W* = 0, *p* < 0.005, *n* = 11), an 843% increase in number, and the number of threatened species (i.e. those classified as critically endangered, endangered, or vulnerable on the International Union for Conservation of Nature [IUCN] Red List) increased significantly (*W* = 0, *p* < 0.005, *n* = 11) from a mean of 27.3 (27.5) to 177.7 (137.4) species, a 551% increase (Table 4). The mean number of bird species that qualified sites as global KBAs remained similar after existing KBAs that had been identified as IBAs were reassessed against the KBA criteria (235.4 [126.8] in 2019 vs. 245.4 [145.9] in 2024; Wilcoxon *W* = 21, *p* > 0.05, *n* = 11). There were large increases in the number of species in other taxonomic groups that qualified sites as KBAs, particularly for plants (Appendix S3). The numbers reflect levels of knowledge, data availability, and species diversity in each class.

**TABLE 4:** Number of taxonomic groups with one or more species meeting global Key Biodiversity Area (KBA) criteria (i.e., for which one or more KBAs have been identified) per country and the total number of species and number of these that are threatened.

Country	Taxonomic groups		Total number of species		Number of threatened species	
	2019	2024	2019	2024	2019	2024
Bolivia	4	25	34	190	18	137
Colombia	6	22	150	481	61	287
Republic of the Congo	2	15	1	66	1	55
D. R. Congo	7	10	52	169	35	94
Ecuador	5	26	143	877	53	408
Gabon	2	28	3	187	1	140
Mozambique	3	38	4	190	2	137
Peru	7	41	185	646	81	297
South Africa	30	49	68	3526	26	359
Uganda	20	23	34	64	21	34
United Arab Emirates	1	3	6	9	1	7
Mean (SD)	7.9(9.0)	25.5(13.5)	61.8(67.0)	582.3(1013.2)	27.3(27.5)	177.7(137.4)

Assessing changes in the numbers of KBAs that qualified for birds (given that many existing KBAs comprised sites originally identified as IBAs), we found that the total number of IBAs meeting global KBA criteria remained stable, changing from 286 sites (mean 28.3% [SD 23.0]) in 2019 to 281 (26.7% [17.4]) in 2024, indicating that assessments of global KBA classification from IBA data were supported and did not change following reassessment. The number of IBAs with a KBA classification of regional or global and regional to be determined decreased from 324 (mean 53.7% [26.5]) to 40 (4.1% [6.2]) across all countries in 2024 (Appendix S4). However, many of these regional and global and regional to be determined KBAs were not delisted but were redelineated or superseded or both through, for example, splitting or merging multiple sites (337 sites, 32.1% [28.0] of all original sites) (Appendix S4). We therefore also evaluated the extent of IBAs in 2019 that was included in the updated KBA network in 2024. On average, 29.6% (26.3) of this extent qualified as global KBAs in 2019 and 2024, 57.3% (20.5) did not qualify as global KBAs in 2019 but did so in 2024, and 2.8% (2.6) qualified as global KBAs in 2019 but not in 2024 (owing to minor boundary revisions to reflect manageability) (Appendix S5). In total, 86.9% of the area of the original IBA network therefore qualified as global KBAs following application of the KBA Standard criteria.

## 4 DISCUSSION

The results across 11 countries show large increases in the number of KBAs and their total extent in each country following relatively comprehensive KBA identification efforts, albeit with considerable variation among countries. On average, the total number of KBAs per country increased by 69.6%, whereas the total extent of KBAs per country increased by 164.2% (Table 1). Fifty‐four percent of KBAs following comprehensive assessment had >50% of their extent outside, rather than inside, the original KBA network. Unsurprisingly, taxonomic representativeness also greatly increased. Large numbers of plant species were added, and there were large proportional increases in the numbers of sites identified for most other taxonomic groups, apart from birds, given the historical focus on this group. Most of the existing KBAs based on IBAs qualified as global KBAs in the final KBA network. They covered 86.9% of the original IBA area (Appendix S5), indicating that the majority of existing KBAs derived from IBA assessments would likely become global KBAs when assessed against the KBA criteria in the KBA Standard.

Some of the expansion of the KBA network overlapped with existing protected areas. The average proportion of KBAs that were completely covered by protected and conserved areas increased from 25.9% to 30.5% of sites. Such newly added KBAs were arguably already recognized as conservation priorities in the relevant countries (although not necessarily for the particular taxa or biodiversity features for which they were found to qualify as KBAs). However, we also found that the percentage of KBAs that had no coverage by protected or conserved areas increased from an average of 32.7% to 41.1% of sites. Of the new KBA area, 47.2% was unprotected (77.2% of the average area increase in a country), indicating that nearly half of new area was newly recognized as important for biodiversity outside existing protected and conserved areas. We conclude that making a comprehensive assessment of KBAs identifies a significant number of sites of biodiversity importance that have not been recognized previously as priorities for conservation.

If these 11 countries were typical of other countries across the world, then the total number of KBAs and the number falling outside the current network would increase substantially once comprehensive assessments have been completed, potentially more than doubling the extent of the global KBA network. However, most of these 11 countries are tropical, highly biodiverse, and support many geographically restricted species (Baisero et al., 2021), which biases the sample. Although similar results are likely from other tropical or biodiversity‐rich countries, the same may not hold elsewhere. The results may be different (i.e., lower coverage by KBAs) in countries at higher latitudes or in less biodiverse areas; therefore, it would be valuable to update our analyses when relatively comprehensive KBA assessments have been undertaken in a sufficient sample of such countries. Canada is close to completing a comprehensive assessment of its KBAs, and Spain, Greece, and Italy are starting national assessments, which would contribute to such an analysis.

The KBAs were estimated to cover about 9% of Earth's terrestrial surface in 2016, and using them to guide protected area expansion could lead to significant benefits for biodiversity conservation (Kullberg et al., 2019). However, the current KBA network does not cover all species or priority areas (Kullberg et al., 2019). Even for birds, existing KBAs do not include any habitat for 29 bird species (mostly taxa that have been recognized taxonomically or classified as threatened since IBA inventories were compiled in the countries in which they occur [Lansley et al., 2025]). The degree to which comprehensively assessed KBA networks in countries perform better than existing KBAs in covering species’ distributions is a priority for future research. Our results showed that total KBA area for our focal countries on average increased from 13.9% in 2019 to 24.6% of land and now covers 27.3% of freshwater water bodies, 23.6% of streams and rivers, and 10.3% of marine systems after comprehensive KBA assessments. Target 3 of the KMGBF aims to conserve 30% of land, freshwater, and seas, especially “areas of particular importance for biodiversity.” KBAs can be used to identify such areas and therefore guide where existing protected areas may be expanded, new protected areas designated, or OECMs recognized in order to reach the 30% target (Plumptre, Baisero, et al., 2024). A small proportion of KBAs may not require designation as protected areas or recognition as OECMs to ensure the persistence of the biodiversity for which they are important (Smith et al., 2019), but these cases are likely to be few. Comprehensive KBA assessments also greatly increase the degree to which the site network is representative across biodiversity. As expected, the number of taxonomic groups with species that qualified sites for KBA status greatly increased following comprehensive assessments. The total number of species that qualified sites as KBAs in the 11 countries increased on average by 843%, and the number of threatened species that qualified sites as KBAs increased by 551%.

The establishment of KBA National Coordination Groups in each of the 11 countries brought together individuals from government agencies, nongovernmental organizations, and academia, and, in some countries, also included Indigenous Peoples’ groups, representatives of businesses, and other stakeholders. The process helped consolidate and synthesize the biodiversity information for each country, yielding benefits for other conservation applications, including planning and policy implementation. Most of the countries’ governments recognized the clear link to national commitments to Target 21 of the KMGBF (with biodiversity data used as the basis of decisions). It also refined the delimitation of existing and new sites, ensuring that manageable areas were more accurately delineated (e.g., South Africa calculated more than a 3‐fold increase in mean number of nodes in shapefiles per unit area). In addition to supporting the achievement of Target 21 of the KMGBF, this compilation of data is valuable because it synthesizes the existing information for a country for policy planning uses. Establishing KBA National Coordination Groups also helped build local support for these sites, including practical conservation outcomes. For instance, in Mozambique, KBAs have been integrated into the national territorial plan, which directs where development, urban and agricultural expansion, and conservation efforts will take place nationwide. They were also a fundamental layer in Mozambique's national marine spatial plan and were pivotal in an official systematic conservation planning exercise to shape the new strategy and action plan for expanding the marine protected area network. Legislation also designates KBAs as protected areas for birds and their habitats, areas to avoid for development, and potential sites for biodiversity offsets for companies that have significant negative residual impacts on biodiversity. Recently, Mozambique's updated Forest Law and its regulation further classify forests in KBAs as conservation forests. In Ecuador, the Consortium of Provincial Governments included KBAs in the *Manual for the Strategic Management of Natural Heritage*. Multiple benefits and contributions to several of the targets of the KMGBF can therefore be derived from making a comprehensive assessment of KBAs in a country.

Although we focused on the species for which sites qualified as KBAs, several countries also applied the KBA criteria for ecosystems and sites of ecological integrity, biodiversity elements that had not been previously considered in KBA identification. However, the KBA criteria relating to ecosystems have not been applied in all countries because of a lack of data on the global extent of ecosystems represented in national ecosystem maps (as such data are needed to apply KBA criteria A2 and B4). There is an urgent need to map ecosystems at level 4 or 5 in the global ecosystem typology (Keith et al., 2022) and make assessments of their IUCN Red List status (Keith et al., 2015; Nicholson et al., 2024) to allow KBA criteria relating to ecosystems to be applied more widely. The Republic of the Congo identified Nouabale Ndoki National Park as the first KBA on the basis of its high ecological integrity (under KBA criterion C), as well as for its importance for threatened species (criterion A1) and geographically restricted species (criterion B1). South Africa applied KBA criteria A2 and B4 to its ecosystems and has been the only country to date to apply KBA criterion E (relating to irreplaceability). Its irreplaceability assessment identified 207 KBAs of the 265 KBAs for the country, but all except 4 of these sites already qualified under other KBA criteria (L. von Staden, personal communication 2025), suggesting that KBA criteria A–D may be effective in identifying sites that also emerge as highly irreplaceable in quantitative analyses.

Our results highlighted that to target expansion of protected areas or OECM networks to meet Target 3 of the KMGBF or to identify expansion areas for biodiversity‐inclusive spatial planning to meet Target 1, it helps to update the national KBA network as part of the process. Identification and reassessment of KBAs can be built into projects or systematic conservation planning exercises (Plumptre, Hayes, et al., 2024) that aim to identify where protected areas should be established or where OECMs should be recognized or that aim to develop biodiversity‐inclusive spatial plans. Doing so will ensure that the sites in a country that are globally significant for biodiversity are identified and delineated so that they can be adequately conserved and incorporated in planning processes. Although the countries that have led the way in identifying and reassessing their KBAs are from Africa, the Middle East, and South America and are mostly tropical countries, previous scoping studies also show the value of making KBA assessments elsewhere, such as in the Global North because sites of global significance may be missed when countries focus on national or regional priorities (Avery et al., 1995; Lim et al., 2023; Spiliopoulou et al., 2024). A comprehensive assessment of KBAs is currently underway in Canada, and KBA National Coordination Groups have been established in several European countries to undertake national assessments (Plumptre, Baisero, et al., 2024). Our results suggest that there is value in all countries updating their KBA network by 2030 as part of their commitment to implement the KMGBF. We encourage all countries that have not yet done so to incorporate this ambition in their planning for Targets 1 and 3, as well as in the ongoing updates of NBSAPs, which are still in development for 80% of countries (CBD, 2022).

## AUTHOR CONTRIBUTIONS

Andrew J. Plumptre drafted the paper and analyzed the results. Zoltan Waliczky, Daniele Baisero, Olivia Crowe, Jeannot Kivono, Cecilia Tobar, Maria Gabriela Toscano, Natalia Boulad, Hugo Costa, Camila Davila, Sophie Dirou, Eleuterio Duarte, Karolina Fierro, Carolina Castellanos‐Castro, Hanna Haddad, Stephen Holness, Fiona Maisels, Daniel Marnewick, Menard Mbende, Maitha Abdulla Al Mheiri, Dissondet Moundzoho, Simon Nampindo, Grace Nangendo, Steeve Ngama, Catherine Numa, Diego Peñaranda, Samridhi Rijal, Manuel Sánchez‐Nivicela, Andrew Skowno, Thomas Starnes, Nicolas Texier, and Lize von Staden were involved in leading comprehensive assessments of KBAs in the 11 countries and contributed to reviewing and editing the manuscript. Andrew J. Plumptre, Zoltan Waliczky, Thomas Starnes, Anne Bowser, Thomas M. Brooks, Gill Bunting, Stuart H. M. Butchart, Neil Cox, Wendy Elliot, Jo Gilbert, Penny Langhammer, Olivier Langrand, Rachel Neugarten, Madhu Rao, Jon Paul Rodriguez, Gina della Togna, Amy Upgren, and Stephen Woodley oversaw the KBA program and contributed to the writing and editing of the paper.

## Supporting information




**Appendix S1**: Key Biodiversity Area (KBA) criteria and thresholds.
**Appendix S2**. Percent coverage of land, freshwater (calculated for area of standing water and length of streams and rivers separately), and seas (in Exclusive Economic Zone) in each country by Key Biodiversity Areas in 2019 and 2024.
**Appendix S3**. Mean number of species by taxon on which qualification as a Key Biodiversity Area were based in 11 countries before (2019) and after (2024) comprehensive KBA assessments.
**Appendix S4**. Changes in the contribution of IBAs to the KBA network following comprehensive KBA assessments.
**Appendix S5**. The resulting percentage areas of the original IBA network in 2019 following comprehensive KBA assessment.

## References

[cobi70151-bib-0001] Avery, M. , Gibbons, D. W. , Porter, R. , Tew, T. , Tucker, G. , & Williams, G. (1995). Revising the British Red Data List for birds: The biological basis of U.K. conservation priorities. Ibis, 137, S232–S239.

[cobi70151-bib-0002] Baisero, D. , Schuster, R. , & Plumptre, A. J. (2021). Redefining and mapping global irreplaceability. Conservation Biology, 36, Article e13806. 34254360 10.1111/cobi.13806

[cobi70151-bib-0003] BirdLife International . (2019). Important Bird and Biodiversity Areas (Version March 2019). https://datazone.birdlife.org/about‐our‐science/ibas

[cobi70151-bib-0004] BirdLife International . (2025). The World Database of Key Biodiversity Areas . Developed by the KBA Partnership: BirdLife International, International Union for the Conservation of Nature, Amphibian Survival Alliance, Conservation International, Critical Ecosystem Partnership Fund, Global Environment Facility, Re:wild, NatureServe, Rainforest Trust, Royal Society for the Protection of Birds, Wildlife Conservation Society and World Wildlife Fund. www.keybiodiversityareas.org

[cobi70151-bib-0005] Butchart, S. H. M. , Scharlemann, J. P. W. , Evans, M. I. , Quader, S. , Aricò, S. , Arinaitwe, J. , Balman, M. , Bennun, L. A. , Bertzky, B. , Besançon, C. , Boucher, T. M. , Brooks, T. M. , Burfield, I. J. , Burgess, N. D. , Chan, S. , Clay, R. P. , Crosby, M. J. , Davidson, N. C. , De Silva, N. , … Woodley, S. (2012). Protecting important sites for biodiversity contributes to meeting global conservation targets. PLoS ONE, 7, Article e32529. 22457717 10.1371/journal.pone.0032529 PMC3310057

[cobi70151-bib-0006] Convention of Biological Diversity (CBD) . (2022). Kunming‐Montreal Global Biodiversity Framework Targets . https://www.cbd.int/gbf/targets

[cobi70151-bib-0007] Convention of Biological Diversity (CBD) . (2024). Recommendations to AHTEG for the Disaggregation of Headline Indicator 3.1 Coverage of Protected and Conserved Areas, to Monitor Status of Inland Waters . https://www.cbd.int/cms/ui/forums/attachment.aspx?id=435&exp=638747886164227711&uid=1&key=23e0c75b539646fab389096e3ba84d6c

[cobi70151-bib-0008] Critical Ecosystem Partnership Fund (CEPF) . (2023). Critical Ecosystem Partnership Report Impact 2001–2023 & 2023 Reports . https://impactreport.cepf.net/

[cobi70151-bib-0009] Darbyshire, I. , Anderson, S. , Asatryan, A. , Byfield, A. , Cheek, M. , Clubbe, C. , Ghrabi, Z. , Harris, T. , Hetubun, C. D. , Kalema, J. , Magassouba, S. , McCarthy, B. , Milliken, W. , de Montmollin, B. , Nic Lughadha, E. , Onana, J.‐M. , Saïdou, D. , Sârbu, A. , Shrestha, K. , & Radford, E. A. (2017). Important Plant Areas: Revised selection criteria for a global approach to plant conservation. Biodiversity & Conservation, 26, 1767–1800.

[cobi70151-bib-0010] Di Marco, M. , Brooks, T. , Cuttelod, A. , Fishpool, L. D. C. , Rondinini, C. , Smith, R. J. , Bennun, L. , Butchart, S. H. M. , Ferrier, S. , Foppen, R. P. B. , Joppa, L. , Juffe‐Bignoli, D. , Knight, A. T. , Lamoreux, J. F. , Langhammer, P. F. , May, I. , Possingham, H. P. , Visconti, P. , Watson, J. E. M. , & Woodley, S. (2016). Quantifying the relative irreplaceability of important bird and biodiversity areas. Conservation Biology, 30, 392–402. 26307601 10.1111/cobi.12609

[cobi70151-bib-0011] Donald, P. , Fishpool, L. , Ajagbe, A. , Bennun, L. , Bunting, G. , Burfield, I. , Butchart, S. H. M. , Capellan, S. , Crosby, M. J. , Dias, M. P. , Diaz, D. , Evans, M. I. , Grimmett, R. , Heath, M. , Jones, V. R. , Lascelles, B. G. , Merriman, J. C. , O'Brien, M. , Ramirez, I. , … Wege, D. C. (2019). Important Bird and Biodiversity Areas (IBAs): The development and characteristics of a global inventory of key sites for biodiversity. Bird Conservation International, 29, 177–198.

[cobi70151-bib-0012] ESRI . (2024). ArcGIS Professional (Release 3.1.3). Author.

[cobi70151-bib-0013] Flanders Marine Institute . (2023). Maritime Boundaries Geodatabase: Maritime Boundaries and Exclusive Economic Zones (200NM), version 12 . 10.14284/632

[cobi70151-bib-0014] Hyde, C. A. , Notarbartolo di Sciara, G. , Sorrentino, L. , Boyd, C. , Finucci, B. , Fowler, S. L. , Kyne, P. M. , Leurs, G. , Simpfendorfer, C. A. , Tetley, M. J. , & Womersley, F. (2022). Putting sharks on the map: A global standard for improving shark area‐based conservation. Frontiers in Marine Science, 9, Article 968853.

[cobi70151-bib-0015] International Union for Conservation of Nature (IUCN) . (2016). A global standard for the identification of Key Biodiversity Areas, Version 1.0. First edition . https://portals.iucn.org/library/node/46259

[cobi70151-bib-0016] KBA Standards and Appeals Committee of IUCN SSC/WCPA . (2022). Guidelines for using a global standard for the identification of Key Biodiversity Areas: Version 1.2 . IUCN.

[cobi70151-bib-0017] Keith, D. A. , Ferrer‐Paris, J. R. , Nicholson, E. , Bishop, M. J. , Polidoro, B. A. , Ramirez‐Lodra, E. , Tozer, M. G. , Nel, J. L. , MacNally, R. , Gregr, E. J. , Watermeyer, K. E. , Essl, F. , Faber‐Langendoen, D. , Franklin, J. , Lehmann, C. E. R. , Etter, A. , Roux, D. J. , Stark, J. S. , Rowland, J. A. , … Kingsford, R. T. (2022). A function‐based typology for Earth's ecosystems. Nature, 610, 513–518. 36224387 10.1038/s41586-022-05318-4 PMC9581774

[cobi70151-bib-0018] Keith, D. A. , Rodríguez, J. P. , Brooks, T. M. , Burgman, M. A. , Barrow, E. G. , Bland, L. , Comer, P. J. , Franklin, J. , Link, J. , McCarthy, M. A. , Miller, R. M. , Murray, N. J. , Nel, J. , Nicholson, E. , Oliveira‐Miranda, M. A. , Regan, T. J. , Rodríguez‐Clark, K. M. , Rouget, M. , & Spalding, M. D. (2015). The IUCN Red List of Ecosystems: Motivations, challenges, and applications. Conservation Letters, 8, 214–226.

[cobi70151-bib-0019] Kullberg, P. , Di Minin, E. , & Moilanen, A. (2019). Using key biodiversity areas to guide effective expansion of the global protected area network. Global Ecology and Conservation, 20, Article e00768. 10.1016/j.gecco.2019.e00768

[cobi70151-bib-0020] Langhammer, P. F. , Bakarr, M. I. , Bennun, L. A. , Brooks, T. M. , Clay, R. P. , Darwall, W. , De Silva, N. , Edgar, G. J. , Eken, G. , Fishpool, L. D. C. , Fonseca, G. A. B. d. , Foster, M. N. , Knox, D. H. , Matiku, P. , Radford, E. A. , Rodrigues, A. S. L. , Salaman, P. , Sechrest, W. , & Tordoff, A. W. (2007). Identification and gap analysis of Key Biodiversity Areas: Targets for comprehensive protected area systems. IUCN.

[cobi70151-bib-0021] Lansley, T. , Crowe, O. , Butchart, S. H. M. , Edwards, D. P. , & Thomas, G. (2025). How effectively do Key Biodiversity Areas represent global avian diversity? Conservation Biology, 2025, Article e70000. 10.1111/cobi.70000 PMC12309649 40033832

[cobi70151-bib-0022] Lehner, B. , Anand, M. , Fluet‐Chouinard, E. , Tan, F. , Aires, F. , Allen, G. H. , Bousquet, P. , Canadell, J. G. , Davidson, N. , Finlayson, C. M. , Gumbricht, T. , Hilarides, L. , Hugelius, G. , Jackson, R. B. , Korver, M. C. , McIntyre, P. B. , Matthews, E. , Nagy, S. , Olefeldt, D. , … Thieme, M. (2025). Mapping the world's inland waters: An upgrade to the Global Lakes and Wetlands Database (GLWD v2). Earth System Science Data, 17, 2277–2329.

[cobi70151-bib-0023] Lim, D. , Starnes, T. , & Plumptre, A. J. (2023). Global priorities for biodiversity conservation in the United Kingdom. Biological Conservation, 277, Article 109798.

[cobi70151-bib-0024] Linke, S. , Lehner, B. , Ouellet Dallaire, C. , Ariwi, J. , Grill, G. , Anand, M. , Beames, P. , Burchard‐Levine, V. , Maxwell, S. , Moidu, H. , Tan, F. , & Thieme, M. (2019). Global hydro‐environmental sub‐basin and river reach characteristics at high spatial resolution. Scientific Data, 6, Article 283. 31819059 10.1038/s41597-019-0300-6 PMC6901482

[cobi70151-bib-0025] Luther, D. , Cooper, W. J. , Wong, J. , Walker, M. , Farinelli, S. , Visseren‐Hamakers, I. , Burfield, I. J. , Simkins, A. , Bunting, G. , Brooks, T. M. , Dicks, K. , Scott, J. , Westrip, J. R. S. , Lamoreux, J. , Parr, M. , de Silva, N. , Foster, M. , Upgren, A. , & Butchart, S. H. M. (2021). Conservation actions benefit the most threatened species: A 13‐year assessment of Alliance for Zero Extinction species. Conservation Science and Practice, 3, Article e510.

[cobi70151-bib-0026] Nicholson, E. , Andrade, A. , Brooks, T. M. , Driver, A. , Ferrer‐Paris, J. R. , Grantham, H. , Gudka, M. , Keith, D. A. , Kontula, T. , Lindgaard, A. , Londono‐Murcia, M. C. , Murray, N. , Raunio, A. , Rowland, J. A. , Sievers, M. , Skowno, A. L. , Stevenson, S. L. , Valderrabano, M. , Vernon, C. M. , … Obura, D. (2024). Roles of the Red List of Ecosystems in the Kunming‐Montreal Global Biodiversity Framework. Nature Ecology and Evolution, 8, 614–621. 38332025 10.1038/s41559-023-02320-5

[cobi70151-bib-0027] Pavón‐Jordán, D. , Abdou, W. , Azafzaf, H. , Balaž, M. , Bino, T. , Borg, J. J. , Božič, L. , Butchart, S. H. M. , Clausen, P. , Sniauksta, L. , Dakki, M. , Devos, K. , Domsa, C. , Encarnaçao, V. , Etayeb, K. , Faragó, S. , Fox, A. D. , Frost, T. , Gaudard, C. , … Lehikoinen, A. (2020). Positive impacts of important bird and biodiversity areas on wintering waterbirds under changing temperatures throughout Europe and North Africa. Biological Conservation, 246, Article 108549.

[cobi70151-bib-0028] Plumptre, A. J. , Baisero, D. , Brooks, T. M. , Buchanan, G. , Butchart, S. H. M. , Bowser, A. , Boyd, C. , Carneiro, A. P. B. , Davies, T. , Elliot, W. , Foster, M. , Langhammer, P. F. , Marnewick, D. , Matiku, P. , McCreless, E. , Raudsepp‐Hearne, C. , Tordoff, A. W. , Azpiroz, A. B. , Trisurat, Y. , & Upgren, A. (2024). Targeting site conservation to increase the effectiveness of new global biodiversity targets. One Earth, 7, 11–17.

[cobi70151-bib-0029] Plumptre, A. J. , Hayes, J. , Baisero, D. , Rose, R. , Holness, S. , von Staden, L. , & Smith, R. J. (2024). The strengths and complementarity of Systematic Conservation Planning and Key Biodiversity Area approaches for spatial planning. Conservation Biology, 39, Article e14400. 39403036 10.1111/cobi.14400 PMC11959315

[cobi70151-bib-0030] Ricketts, T. H. , Dinerstein, E. , Boucher, T. , Brooks, T. M. , Butchart, S. H. M. , Hoffmann, M. , Lamoreux, J. M. , Morrison, J. , Parr, M. , Pilgrim, J. D. , Rodrigues, A. S. L. , Sechrest, W. , Wallace, G. E. , Berlin, K. , Bielby, J. , Burgess, N. D. , Church, D. R. , Cox, N. , Knox, D. , … Wikramanayake, E. (2005). Pinpointing and preventing imminent extinctions. Proceedings of the National Academy of Sciences of the United States of America, 102, 18497–18501. 16344485 10.1073/pnas.0509060102 PMC1311739

[cobi70151-bib-0031] Smith, R. J. , Bennun, L. , Brooks, T. M. , Butchart, S. H. , Cuttelod, A. , Di Marco, M. , Ferrier, S. , Fishpool, L. D. , Joppa, L. , Juffe‐Bignoli, D. , Knight, A. T. , Lamoreux, J. F. , Langhammer, P. , Possingham, H. P. , Rondinini, C. , Visconti, P. , Watson, J. E. , Woodley, S. , Boitani, L. , … Scaramuzza, C. A. d. M. (2019). Synergies between the key biodiversity area and systematic conservation planning approaches. Conservation Letters, 12, Article e12625.

[cobi70151-bib-0032] Spiliopoulou, K. , Rigal, F. , Plumptre, A. J. , Trigas, P. , Paragamian, K. , Hochkirch, A. , Lymberakis, P. , Portolou, D. , Stoumboudi, M. T. , & Triantis, K. A. (2024). KBAscope: Key biodiversity area identification in R. Ecography, 2024, Article e07061.

[cobi70151-bib-0033] Tetley, M. J. , Braulik, G. T. , Lanfredi, C. , Minton, G. , Panigada, S. , Politi, E. , Zanardelli, M. , Notarbartolo di Sciara, G. , & Hoyt, E. (2022). The Important Marine Mammal Area network: A tool for systematic spatial planning in response to the marine mammal habitat conservation crisis. Frontiers in Marine Science, 9, Article 841789.

[cobi70151-bib-0036] United Nations . (2025). The Sustainable Development Goals Report 2025 . United Nations, New York, USA.

[cobi70151-bib-0034] Zar, J. H. (1984). Biostatistical analysis (2nd ed.). Prentice‐Hall International.

