# Supplementary material for: Changes in Key Biodiversity Area networks following national comprehensive assessments
Source: Conserv Biol. 2025 Sep 30;40(1):e70151. doi: 10.1111/cobi.70151 (PMC12856777; doi:10.1111/cobi.70151)
Supplement: Supplementary file 1 — Appendix S1: Key Biodiversity Area (KBA) criteria and thresholds. Appendix S2. Percent coverage of land, freshwater (calculated for area of standing water and length of streams and rivers separately), and seas (in Exclusive Economic Zone) in each country by Key Biodiversity Areas in 2019 and 2024. Appendix S3. Mean number of species by taxon on which qualification as a Key Biodiversity Area were based in 11 countries before (2019) and after (2024) comprehensive KBA assessments. Appendix S4. Changes in the contribution of IBAs to the KBA network following comprehensive KBA assessments. Appendix S5. The resulting percentage areas of the original IBA network in 2019 following comprehensive KBA assessment. [file COBI-40-e70151-s001.docx]

**Supporting information**

**Appendix S1:** Key Biodiversity Area (KBA) criteria and thresholds.

| A. Threatened biodiversity | | |  |
| --- | --- | --- | --- |
| A1 Threatened species | | Assessment parameters |  |
| A1a | ≥0.5% of global population size and ≥5 reproductive units (RU) of a CR/EN species | (i) number of mature individuals  (ii) area of occupancy  (iii) extent of suitable habitat  (iv) range  (v) number of localities  (vi) distinct genetic diversity |  |
| A1b | ≥1% of global population size and ≥10 RU of a VU species |  |  |
| A1c | ≥0.1% of global population size and ≥5 RU of a species listed as CR/EN due only to past/current decline [= Red List A only, but not A3 only] |  |  |
| A1d | ≥0.2% of global population size and ≥10 RU of a species listed as VU due only to past/current decline [= Red List A only, but not A3 only] |  |  |
| A1e | Effectively the entire population size of a CR/EN species |  |  |
| A2 Threatened ecosystems | | |  |
| A2a | ≥5% of global extent of a CR or EN ecosystem | |  |
| A2b | ≥10% of global extent of a VU ecosystem | |  |
| B. Geographically restricted biodiversity | | |  |
| B1. Individual geographically restricted species | ≥10% of global population size and ≥10 RU of any species | (i) number of mature individuals  (ii) area of occupancy  (iii) extent of suitable habitat  (iv) range  (v) number of localities  (vi) distinct genetic diversity | |
| B2. Co-occurring geographically restricted species | ≥1% of global population size of each of a number of restricted-range species in a taxonomic group: ≥2 species or 0.02% of the total number of species in the taxonomic group, whichever is larger |  | |
| B3. Geographically restricted assemblages | | |  |
| B3a | ≥0.5% of global population size of each of a number of ecoregion-restricted species in a taxonomic group: ≥5 species or 10% of the species restricted to ecoregion, whichever is larger | (i) number of mature individuals  (ii) area of occupancy  (iii) extent of suitable habitat  (iv) range  (v) number of localities | |
| B3b | ≥5 RU of ≥5 bioregion-restricted species or ≥5 RU of 30% of the bioregion-restricted species known from the country, whichever is larger |  | |
| B3c | Site is part of the globally most important 5% of occupied habitat for ≥5 species in the taxonomic group | (i) relative density of mature individuals  (ii) relative abundance of mature individuals | |
| B4. Geographically restricted ecosystem | | |  |
|  | ≥20% of the global extent of an ecosystem |  | |
| C. Ecological integrity | | |  |
|  | Site is one of ≤2 per ecoregion with wholly intact ecological communities | composition and abundance of species and interactions | |
| D. Biological processes | | |  |
| D1. Demographic aggregations | | |  |
| D1a | ≥1% of global population size of a species, over a season, and during ≥1 key stage in life cycle | number of mature individuals |  |
| D1b | Site is among largest 10 aggregations of the species | number of mature individuals |  |
| D2. Ecological refugia | ≥10% of global population during periods of environmental stress | number of mature individuals |  |
| D3. Recruitment sources | Produces propagules, larvae or juveniles maintaining ≥10% of global population size | number of mature individuals |  |
| E. Irreplaceability through quantitative analysis | | |  |

Source: IUCN (2016). *A Global Standard for the Identification*, Version 1.0, 1st edn. Gland, Switzerland: IUCN.

**Appendix S2.** Percent coverage of land, freshwater (calculated for area of standing water and length of streams and rivers separately), and seas (in Exclusive Economic Zone) in each country by Key Biodiversity Areas in 2019 and 2024.

| **Country** | **Coverage of land (%)** | | **Coverage of freshwater (%)** | | | | **Coverage of seas (%)** | | |
| --- | --- | --- | --- | --- | --- | --- | --- | --- | --- |
|  | **2019** | **2024** | **Coverage of standing water area (%)** | | **Coverage of stream-river length (%)** | | **2019** | **2024** |  |
|  |  |  | **2019** | **2024** | **2019** | **2024** |  |  |  |
| **Bolivia** | 21.2 | 33.3 | 30.0 | 47.2 | 20.8 | 34.3 | -^a^ | -^a^ |  |
| **Colombia** | 8.4 | 12.7 | 6.8 | 9.8 | 7.5 | 11.9 | 9.9 | 10.0 |  |
| **Republic of Congo** | 19.8 | 26.9 | 12.9 | 13.0 | 7.2 | 16.2 | 0.1 | 13.8 |  |
| **D. R. Congo** | 7.0 | 15.9 | 41.0 | 48.4 | 38.3 | 55.2 | 0.0 | 0.0 |  |
| **Ecuador** | 37.1 | 55.6 | 17.0 | 50.1 | 10.1 | 24.4 | 0.5 | 23.2 |  |
| **Gabon** | 10.4 | 24.5 | 9.9 | 23.5 | 3.1 | 17.7 | 0.2 | 23.2 |  |
| **Mozambique** | 3.0 | 17.1 | 20.7 | 44.2 | 18.1 | 37.3 | 0.2 | 1.1 |  |
| **Peru** | 17.6 | 35.4 | 26.5 | 17.5 | 19.1 | 25.5 | 0.5 | 0.5 |  |
| **South Africa** | 16.2 | 29.2 | 19.4 | 26.7 | 15.9 | 27.6 | ^a^ | ^a^ |  |
| **Uganda** | 8.4 | 9.7 | 6.8 | 6.7 | 10.3 | 10.2 | ^a^ | ^a^ |  |
| **United Arab Emirates** | 3.6 | 10.7 | 6.3 ^b^ | 12.9 ^b^ | 3.3 ^b^ | 10.2 ^b^ | 1.3 | 10.5 |  |
| **Mean**  **(Stand. Dev.)** | **13.9**  **(9.9)** | **24.6**  **(13.7)** | **17.9**  **(11.1)** | **27.3**  **(17.0)** | **13.3**  **(10.2)** | **23.6**  **(13.8)** | **1.6**  **(3.4)** | **10.3**  **(9.5)** |  |

^a^ Uganda and Bolivia do not have marine territory and South Africa has not yet applied the KBA criteria to their marine territory in their recent assessment but plan to do so,

^b^ Values based on global estimates of water, and seasonal and permanent water are not separated. Many waterbodies are highly seasonal.

**Appendix S3.** Mean number of species by taxon on which qualification as a Key Biodiversity Area were based in 11 countries before (2019) and after (2024) comprehensive KBA assessments.


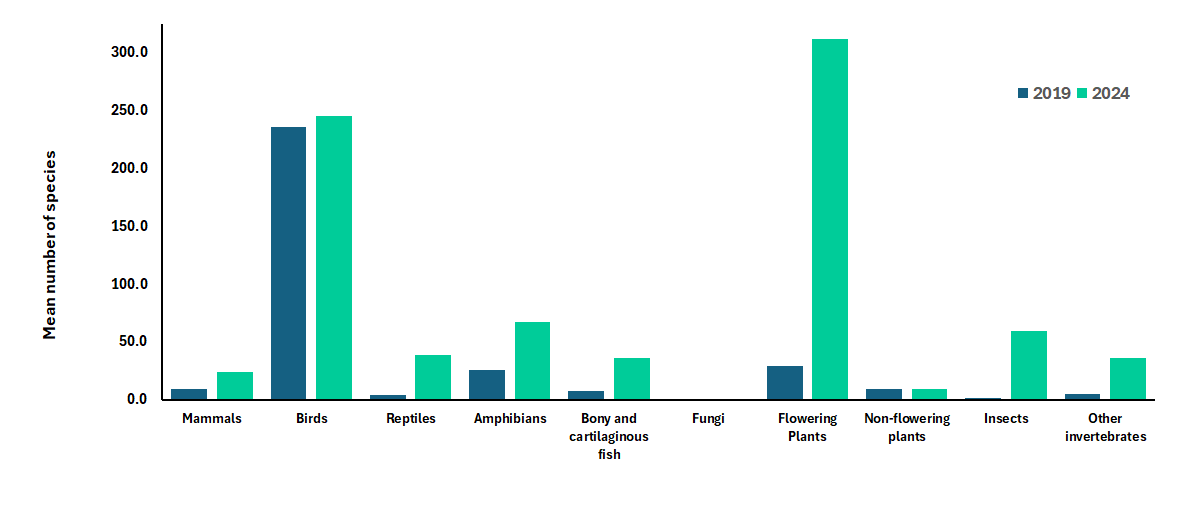


**Appendix S4**. Changes in the contribution of IBAs to the KBA network following comprehensive KBA assessments.

| **Country** | **Percentage of sites that qualified as global KBAs and IBAs for birds** | | **Percentage of sites that were IBAs but did not qualify as global KBAs** | | **Percentage of sites that were not IBAs** | | **Percentage of all original sites that were superseded** |
| --- | --- | --- | --- | --- | --- | --- | --- |
|  | **2019** | **2024*** | **2019** | **2024*** | **2019** | **2024*** |  |
| **Bolivia** | 50.9 | 35.2 | 33.9 | 1.1 | 15.3 | 63.7 | 33.9 |
| **Colombia** | 57.1 | 55.9 | 24.2 | 8.1 | 18.8 | 36.0 | 10.7 |
| **Republic of Congo** | 0.0 | 40.0 | 90.0 | 0.0 | 10.0 | 60.0 | 10.0 |
| **D.R. Congo** | 33.3 | 34.8 | 45.8 | 2.2 | 20.8 | 60.9 | 8.3 |
| **Ecuador** | 50.8 | 27.4 | 37.7 | 0.0 | 11.5 | 72.6 | 66.4 |
| **Gabon** | 0.0 | 8.6 | 87.5 | 0.0 | 12.5 | 91.4 | 62.5 |
| **Mozambique** | 28.6 | 24.3 | 47.6 | 10.8 | 23.8 | 59.5 | 19.1 |
| **Peru** | 45.8 | 26.5 | 29.0 | 4.3 | 25.2 | 69.2 | 33.6 |
| **South Africa** | 4.1 | 1.5 | 54.7 | 0.0 | 41.2 | 98.5 | 87.7 |
| **Uganda** | 40.5 | 39.7 | 40.5 | 19.0 | 19.1 | 34.5 | 4.8 |
| **United Arab Emirates** | 0.0 | 0.0 | 100.0 | 0.0 | 0.0 | 100.0 | 16.7 |
| **Mean**  **(Stand. Dev.)** | **28.3**  (23.0) | **26.7**  (17.4) | **53.7**  (26.5) | **4.1**  (6.2) | **18.0**  (10.5) | **67.8**  (22.1) | **32.1**  (28.0) |

* Note that IBA status was not reassessed in 2024, and it is likely that many sites qualifying as Global KBAs in 2024 (whether for birds or other taxa, or qualifying under other KBA criteria) would qualify as IBAs if reassessed.

**Appendix S5**. The resulting percentage areas of the original IBA network in 2019 following comprehensive KBA assessment.

| **Country** | **Percentage area of IBAs in 2019 that qualified as KBAs in 2024.** | | **Percentage area of sites that qualified as IBAs in 2019 that are no longer KBAs (owing to revisions in delineation or delisting)** | |
| --- | --- | --- | --- | --- |
|  | **Global KBA and IBA in 2019** | **IBA only in 2019** | **Global KBA and IBA in 2019** | **IBA only in 2019** |
| **Bolivia** | 35.4 | 54.2 | 3.2 | 7.2 |
| **Colombia** | 33.1 | 63.2 | 2.3 | 1.3 |
| **Republic of Congo** | 0.0 | 92.9 | 0.0 | 7.1 |
| **D.R. Congo** | 21.3 | 56.0 | 4.8 | 17.9 |
| **Ecuador** | 42.7 | 49.3 | 4.0 | 4.0 |
| **Gabon** | 0.0 | 73.7 | 0.0 | 26.3 |
| **Mozambique** | 34.8 | 57.4 | 7.7 | 0.1 |
| **Peru** | 65.7 | 33.7 | 0.2 | 0.4 |
| **South Africa** | 14.1 | 54.4 | 5.4 | 26.1 |
| **Uganda** | 78.8 | 17.6 | 3.1 | 0.5 |
| **United Arab Emirates** | 0.0 | 77.7 | 0.0 | 22.3 |
| **Mean**  **(Stand. Dev.)** | **29.6**  (26.3) | **57.3**  (20.5) | **2.8**  (2.6) | **10.3**  (10.7) |
